# Single access laparoscopic nephrectomy

**DOI:** 10.4103/0970-1591.44247

**Published:** 2008

**Authors:** Jay D. Raman, Jeffrey A. Cadeddu

**Affiliations:** Department of Urology, University of Texas Southwestern Medical Center, Dallas, TX, USA

**Keywords:** Keyhole surgery, kidney, minimally invasive surgery, notes, single port

## Abstract

Laparoscopic nephrectomy has assumed a central role in the management of benign and malignant kidney diseases. While laparoscopy is less morbid than open surgery, it still requires several incisions each at least 1-2 cm in length. Each incision carries morbidity risks of bleeding, hernia and/or internal organ damage, and incrementally decreases cosmesis. An alternative to conventional laparoscopy is single access or keyhole surgery, which utilizes magnetic anchoring and guidance system (MAGS) technology or articulating laparoscopic instruments. These technical innovations obviate the need to externally space trocars for triangulation, thus allowing for the creation of a small, solitary portal of entry into the abdomen. Laboratory and early clinical series demonstrate feasibility as well as safe and successful completion of keyhole nephrectomy. Future work is necessary to improve existing instrumentation, increase clinical experience, assess benefits of this surgical approach, and explore other potential applications for this technique.

## INTRODUCTION

Open nephrectomy has historically been the gold standard therapy for the management of benign and malignant kidney disease. Despite evolution of open kidney surgery, considerable morbidity and delayed convalescence occurs from the muscle splitting flank incision. Since the first laparoscopic nephrectomy by Clayman and colleagues in 1991, minimally invasive urologic surgery has gained significant momentum.[1] Advantages of laparoscopic nephrectomy in comparison to open surgery are well established.[2,3] While the laparoscopic approach decreases surgical morbidity, it still requires three to four incisions each at least 1-2 cm in length. In addition, each working port carries morbidity risks of bleeding, hernia and/or internal organ damage, and incrementally decreases cosmesis.[4,5] Cosmesis is particularly important in procedures on pediatric patents and is also demanded by sophisticated adult patients.[6] In an effort to reduce these sequelae some have advocated specimen morcellation and transvaginal extraction of nephrectomy specimens.[7,8]

## NOTES

Natural orifice transluminal endoscopic surgery (NOTES) has been described as the next surgical frontier with the objective of incision-free abdominal surgery. NOTES approaches abdominal surgery through natural orifices (mouth, vagina, and rectum) thus obviating external abdominal scars. Contemporary laboratory investigation is investigating the infectious and immunologic implications of NOTES. Indeed, the concept of a purposeful viscerotomy either using the gastric or vaginal route raises concerns of intrabdominal contamination. The immunologic impacts, however, may actually be favorable for NOTES surgery. McGee and colleagues demonstrated lower levels of tumor necrosis factor-α (TNF-α) after NOTES peritoneoscopy compared to conventional laparoscopic exploration.[9] Hence, NOTES may in fact contribute to less impairment of the peritoneal immune system with potentially improved infectious outcomes.

Animal models have been used to demonstrate the potential applications of NOTES, including transgastric and transvesical peritoneoscopy, transvaginal tubal ligation, hysterectomy, and cholecystectomy.[10] Preliminary clinical series in human patients with transvaginal NOTES cholecystectomy also appear promising.[11] With regards to NOTES renal surgery, Gettman and colleagues reported in 2002 on the successful completion of six laparoscopic transvaginal nephrectomies using conventional instrumentation in a porcine model.[12] However, they noted that limitations of the laparoscopic instrumentation made the procedure “cumbersome and time consuming.” More recently, Clayman *et al*. presented their experience with single-port NOTES transvaginal nephrectomy and encountered similar difficulty until a purpose-built multilumen operating platform was utilized.[13] Even with this improvement in technology, the total operative time was 300 min.

Potential drawbacks to NOTES nephrectomy are not insignificant. Operative duration is longer than conventional laparoscopy, specialized equipment is necessary, and there is a steep learning curve.

## SINGLE ACCESS “KEYHOLE” NEPHRECTOMY

Triangulation is one of the fundamental concepts of conventional laparoscopic surgery. An alternative to conventional laparoscopy and NOTES is single access or keyhole surgery utilizing a magnetically anchored guidance system, articulating laparoscopic instruments, and/or specialized trocars.

### Magnetic anchoring and guidance system (MAGS)

Park and colleagues have recently developed a novel adjunct laparoscopic system consisting of a moveable, “lockable” platform that is positioned intraabdominally and stabilized by an external permanent magnet on the abdominal skin.[14] MAGS can be used to actively control an intraabdominal camera and working instruments introduced through a single trocar. In fact, Zeltser *et al*. have subsequently described the first successful completion of two nonsurvival porcine nephrectomies via a single 15 mm transumbilical trocar using a prototype MAGS camera and a magnetically anchored robotic arm cauterizer.[15]

Prior to widespread adoption of the MAGS platform, both clinical and engineering limitations must be addressed. Surgeons must become familiar with the MAGS components both in a dry laboratory and in animal models. As with all new technology, there will be a learning curve and it will be incumbent on surgeons to develop new “MAGS techniques” by modifying traditional laparoscopic modalities. The coupling strength of magnetics (electromagnetic or permanent magnets) decreases as a decaying exponential with respect to the distance between the source magnet and its target. Currently, tissue thicknesses in excess of 1.5 cm limit the effectiveness of the paddle retractor, while the camera can be supported up to tissue thicknesses of 2.5 cm. As such, present day clinical utilization of MAGS technology would be restricted to thin or pediatric patients. Future directions are needed to develop electromagnets capable of generating stronger magnetic fields. Finally, additional work is needed to develop a more robust MAGS camera system. Current laboratory work has been limited by fogging of the camera and a lack of sufficient lighting (despite on-board LED). Some cases have required laparoscope and flexible endoscope assistance for visualization. Purpose-built modifications in camera design are necessary to obviate the need for additional lighting sources.

MAGS technology is still currently in evolution and is not commercially available for clinical uses.

### Articulating instrumentation

**Figure 1:**
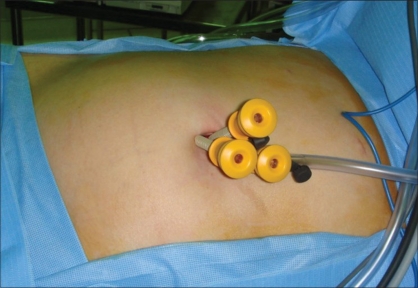
Keyhole umbilical nephrectomy utilizing three adjacent 5-mm trocars

An alternative to MAGS for single access surgery involves using articulating instrumentation via a single large caliber trocar or small, adjacent trocars. Advances in technology have led to the development of new laparoscopic access ports (R-Port, Advanced Surgical Concepts, Wicklow, Ireland and Uni-X Single Port, PNavel Systems, Cleveland, OH, USA) capable of allowing multiple instruments to be inserted through different cannulas of a single port. Alternatively, adjacent 5 mm trocars can be utilized with skin incisions connected at the time of specimen extraction [Figure 1]. The latter may in fact accomplish the same goal of single incision surgery without the incremental cost of multiaccess port technology.

Articulating instrumentation allows for triangulation to occur intracorporeally despite trocars being adjacent to one another through the same skin incision. Currently, articulating laparoscopic graspers (Real Hand, Novare Surgical Systems, Cupertino, CA, USA and Autonomy Laparo-angle, Cambridge Endo, Framingham, MA, USA), endoshears (Cambridge Endo), and laparoscopic needle drivers (Cambridge Endo) are commercially available for clinical use. Optimal use of instrumentation requires crossing intracorporeally such that tissue manipulation, traction, and cautery are performed with the contralateral hand compared with conventional laparoscopy. Such differences and collision of instrumentation creates an inherent learning curve during initial procedures; though, this curve is significantly less steep than for NOTES surgery.

In conjunction with articulating instrumentation, the development of novel intrabdominal retractors will further facilitate evolution of laparoscopic procedures. One such device is the padron endoscopic exposing retractor (PEER) which can be deployed intracorporeally through a 5- or 10-mm port. Adequate and stable positioning of the intrabdominal retractor provides excellent and secure visualization of the operative field during laparoscopic procedures.[16]

**Figure 2:**
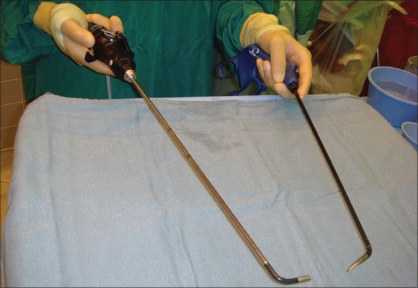
Left hand with articulating laparoscopic grasper (Real Hand, Novare Surgical Systems, Cupertino, CA, USA) and right hand holding 5-mm deflectable tip video laparoscope (Olympus, Orangeburg, NY, USA)

Another important component is the selection of an appropriate laparoscope to optimize visualization while minimizing collision with working instruments. Anecdotally, we have found that laparoscopes using right angle light sources to be problematic due to collision with working instruments. More recently, we have used 45° 5-mm rigid laparoscope with an end light source (Karl Storz, Tuttlingen, Germany) or a 5-mm deflectable tip video laparoscope (Olympus, Orangeburg, NY, USA) [Figure 2].

## INITIAL CLINICAL EXPERIENCE

**Figure 3:**
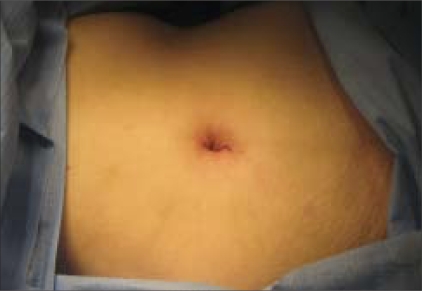
Two centimeters periumbilical incision after keyhole nephrectomy for a nonfunctional kidney removed by specimen morcellation

Early laboratory and clinical experience with single access umbilical nephrectomy with articulating instrumentation is promising. Raman and colleagues recently reported their initial experience with keyhole nephrectomy in a porcine model and in human subjects.[17] In their series, keyhole nephrectomy was successfully completed in all eight porcine renal units and in all three human subjects. The mean operative time for the porcine nephrectomies was 49 min (range, 20-85), with a mean blood loss of 20 cc (range, 5-100). Incision size ranged from 3 to 5 cm. The mean operative time for the human nephrectomy cases was 133 min (range, 90-160). Estimated blood loss was 30 cc, and the kidneys were extracted through a solitary 2-4.5 cm periumbilical incision [Figure 3]. There were no perioperative complications, and all three patients were discharged on hospital day 2. Subsequent clinical work from this group as well as other small clinical series from several institutions have similarly supported the feasibility, safety, and successful completion of single access nephrectomy.[18–20]

The attractiveness of keyhole umbilical nephrectomy is multifaceted. First, it improves cosmesis by allowing for a single umbilical incision. Second, it is within a surgeon's comfort range since specimen extraction occurs via the abdomen. This may be a significant consideration as vaginal or gastric incisions may present complications. Third, the learning curve appears to be much shorter than for NOTES. This is attributable to instrumentation that is similar to conventional laparoscopic devices. Finally, keyhole umbilical surgery provides a “familiar” anatomical view of the kidney which may be lost during the evolution of transvaginal, transgastric, or transcolonic surgery.

Although the early experience for keyhole umbilical surgery is promising, experienced laparoscopic skill is essential for the safe and effective completion of the procedure. As such, coordination between the surgeon and the camera driver is essential. Single port umbilical surgery does permit the introduction of other transabdominal conventional laparoscopic ports to aid completion of the surgical procedure if failure of progression occurs.

## FUTURE DIRECTIONS

Future work with keyhole umbilical surgery is multifaceted. Evolution of MAGS technology and articulating instrumentation are necessary to improve the ergonomics and visualization of the surgical procedure. Proponents of single access surgery suggest that in addition to benefits in cosmesis, there is the possibility of less perioperative pain and morbidity. Comparison of short-term measures of convalescence to that of NOTES and traditional laparoscopic surgery are needed to better address this issue. To date, in addition to our experience with keyhole nephrectomy, we have also completed three single access pyeloplasties and a single access adrenalecomy.[19] Other groups have reported similar success with such surgery, as well as with laparoscopic cryoablation and sacrocolpopexy.[20,21] Future endeavors may involve more complicated operative procedures such as laparoscopic partial nephrectomy and prostatectomy.

## CONCLUSION

Single access umbilical nephrectomy is feasible. Using varied instrumentation and technology, several groups have demonstrated safe and successful completion in both a porcine model and in human patients. Future work will need to assess benefits of keyhole surgery and explore other potential applications for this novel approach.
